# Contemporary Biomarkers in Pulmonary Embolism Diagnosis: Moving beyond D-Dimers

**DOI:** 10.3390/jpm12101604

**Published:** 2022-09-29

**Authors:** Androniki Gkana, Androniki Papadopoulou, Maria Mermiri, Eleftherios Beltsios, Dimitrios Chatzis, Foteini Malli, Antonis Adamou, Konstantinos Gourgoulianis, Georgios Mavrovounis, Ioannis Pantazopoulos

**Affiliations:** 1 Department of Emergency Medicine, Faculty of Medicine, University of Thessaly, Biopolis, 41110 Larissa, Greece; 2 Department of Anaesthesiology, Faculty of Medicine, University of Thessaly, Biopolis, 41110 Larissa, Greece; 3 Department of Thoracic and Cardiovascular Surgery, West Germany Heart Center, University Clinic Essen, 45147 Essen, Germany; 4 School of Medicine, European University of Cyprus, 2404 Nicosia, Cyprus; 5 Department of Respiratory Medicine, Faculty of Medicine, University of Thessaly, Biopolis, 41110 Larissa, Greece

**Keywords:** pulmonary embolism, diagnosis, D-dimers, biomarkers, ischemia modified albumin, microparticles, microRNAs, Factor VIII

## Abstract

Pulmonary embolism (PE) is a rather common cardiovascular disorder constituting one of the major manifestations of venous thromboembolism (VTE). It is associated with high mortality and substantial recurrence rates, and its diagnosis may be challenging, especially in patients with respiratory comorbidities. Therefore, providing a prompt and accurate diagnosis for PE through developing highly sensitive and specific diagnostic algorithms would be of paramount importance. There is sound evidence supporting the use of biomarkers to enhance the diagnosis and predict the recurrence risk in patients with PE. Therefore, several novel biomarkers, such as factor VIII, Ischemia Modified Albumin, and fibrinogen, as well as several MicroRNAs and microparticles, have been investigated for the diagnosis of this clinical entity. The present review targets to comprehensively present the literature regarding the novel diagnostic biomarkers for PE, as well as to discuss the evidence for their use in daily routine.

## 1. Introduction

Pulmonary embolism (PE) is a relatively common cardiovascular disorder that constitutes one of the two major manifestations of venous thromboembolism (VTE). PE may be associated with high mortality, especially in untreated cases; undiagnosed PE carries a 30% mortality rate, which falls to 8% when diagnosed [1]. PE diagnosis may be challenging, especially in patients with respiratory comorbidities [1]. Therefore, it is crucial to provide a prompt and accurate diagnosis for PE by developing highly sensitive and specific diagnostic algorithms.

The diagnosis of the disease is based on the individual patient’s clinical probability for PE combined with laboratory and non-invasive imaging methods, mainly Computed Tomography Pulmonary Angiography (CTPA), which serves as the gold standard [2]. Since PE pathophysiology is rather complex, incorporating both thrombotic and inflammatory components, a plethora of “novel” biomarkers for PE diagnosis is currently under investigation with the ultimate goal to increase diagnostic accuracy, leading to the effective and appropriate management of this potentially lethal clinical entity.

The purpose of the present narrative review is to discuss some of the novel diagnostic biomarkers for PE and illustrate the evidence for their use in everyday clinical practice. Furthermore, we provide an updated review of the contemporary approaches concerning D-dimer testing for the diagnosis of PE.

## 2. Methods

To identify relevant articles for the present literature review, we performed an electronic search of the PubMed (MEDLINE), Google Scholar, and Scopus databases. We used the following keywords combined with the Boolean operators “AND” and “OR”, as appropriate: “Pulmonary Embolism”, “Diagnosis”, “D-dimers”, “Biomarkers”, “Ischemia Modified Albumin”, “MicroRNAs”, “Fibrinogen”, “Factor VIII”, and “Microparticles”. The results were limited to those written in English. The last literature search was performed on 1 March 2022. Three independent investigators (A.G., A.P., M.M) screened the titles and abstracts to select potentially relevant articles for inclusion. The full text of the selected articles was thoroughly examined, and the studies presenting original data on PE diagnosis and different biomarkers were finally included.

## 3. Fibrinogen

Fibrinogen is a large, complex, fibrous glycoprotein, which is converted into fibrin during the coagulation cascade, yielding the fibrin clot for hemostasis [3,4]. Moreover, fibrinogen is produced as an acute phase reactant by the liver in response to inflammation or ischemia. The cleaving of fibrin by plasmin results in the production of D-dimers, which represent the expression of fibrin degradation occurring during the fibrinolytic activity of clot breakdown [5]. Due to its nature as an acute phase reactant, as well as a significant part of the coagulation cascade, the measurement of fibrinogen levels combined with D-dimer levels has been proposed as a valuable diagnostic tool for the diagnosis of PE.

A prospective study assessed the D-dimer and fibrinogen levels in 191 outpatients with suspected PE and observed that patients suffering from PE had a lower fibrinogen and higher D-dimer/fibrinogen (D/F) ratio versus those without PE [6]. The inverse relation of D-dimer and fibrinogen is indicative of activated coagulation leading to fibrinogen consumption and the simultaneous activation of endogenous fibrinolysis, resulting in D-dimer elevation. Moreover, low fibrinogen levels can be explained by the impaired fibrinogen synthesis due to liver congestion caused by right ventricular failure in patients with PE. At the cut-off point of 100% specificity, the true PPV of D/F ratio > 1.04 × 103 was approximately twice as high when compared with D-dimer > 7000 mg/L (57.6% vs. 29.4%). As such, the authors supported that a D/F ratio > 1000 is highly specific for acute PE and might be used as a “rule in” test. [6]. In the same context, Kara et al. [4] demonstrated that patients with PE had q significantly increased D/F ratio compared to controls. However, this change was attributed to increased D-dimer levels, since fibrinogen did not differ between groups. Importantly, the D/F ratio displayed greater specificity than D-dimer levels alone for PE diagnosis (37% vs. 27%, respectively) [4].

Interestingly, in a large Danish study of 77,608 individuals, high fibrinogen levels were observed in patients with PE in combination with DVT; fibrinogen levels ≥ 4.6 g/L were associated with a multivariate-adjusted odds ratio of 2.1 [7]. On the other hand, to make matters more complicated, in a prospective study of 40 PE patients, one-third of the patients had a fibrinogen level out of the normal range and the study did not reveal lower fibrinogen levels in patients with a positive D-dimer test [8].

Another argument that has been raised is whether the D/F ratio may be utilized for the diagnosis of PE in specific clinical settings, such as Intensive Care Unit (ICU) patients. Critically ill patients may have elevated D-dimer and low fibrinogen levels due to several factors, such as infections, malignancies, or severe cardiac or respiratory diseases, rendering them unreliable for the diagnosis of PE [9,10]. In these instances, the D/F ratio may be preferable instead. Indeed, Hajsadeghi et al. [10] suggested that the D/F ratio in ICU patients was significantly higher when PE was present, having almost the same AUC with D-dimer for diagnosing patients with PE (0.710 vs. 0.714 for D/F ratio and D-dimers, respectively), while in contrast, the fibrinogen levels did not differ significantly (536.73 ± 186.32 vs. 586.33 ± 211.06, *p* = 0.298). More specifically, a D/F cut-off ratio of 0.233 × 10^−3^ had the highest accuracy in the diagnosis of PE in an ICU setting (sensitivity 70%, specificity 67.1%) [10].

In conclusion, the utility of the D-dimer/fibrinogen ratio as a diagnostic tool for the diagnosis of PE, although promising, remains controversial. The data found in the literature are not conclusive and are derived from small cohorts. One has to take into account that the aforementioned data lack external validation and cannot be safely applied in clinical practice before further larger randomized clinical trials confirm their findings [4,6,10]. Table 1 summarized the available data on the use of fibrinogen in the diagnosis of PE.

## 4. Ischemia-Modified Albumin (IMA)

Ischemia-Modified Albumin (IMA) is a molecule formed by the modification of albumin by reactive oxygen radicals [11]. Human serum albumins consist of 585 amino acids and the first 3 amino acids in the N-terminus, Asp-Ala-His, constitute a specific binding site for transition metals, which is susceptible to degradation. IMA is formed through the modification of this protein region due to the effect of reactive oxygen radicals produced by ischemia [12]. Serum IMA levels may increase in several acute conditions, namely, acute coronary syndrome, cardiac arrest, stroke, and mesenteric ischemia [11,13,14].

Published data from small cohort studies suggest that IMA levels are higher in PE patients vs. controls, and thus IMA has been proposed as a potential diagnostic biomarker for PE [15]. Turedi et al. [16] studied 30 PE patients and 30 healthy volunteers and demonstrated a statistically significant increase in serum IMA levels above 0.540 Absorbance Units in 97.7% of PE patients. In line with the aforementioned findings, a serum IMA above 0.4 had a sensitivity of 53.8% and specificity of 89.6% for PE, and the IMA levels were positively correlated with shock index and heart rate but failed to predict RV dysfunction in an experimental animal study [11]. A consequent study suggested that the IMA levels, in combination with clinical probability scores, had similar negative predictive value (NPV) and sensitivity to D-dimer testing [15]. However, IMA had a greater positive predictive value (PPV) compared to D-dimer (79.4% vs. 69.4%) but was not high enough to confirm the diagnosis of PE without additional investigation [15].

Despite that the aforementioned data favor a role of IMA in PE diagnosis, they should be interpreted with caution since they derive from small retrospective cohorts (i.e., patients were not followed-up for subsequent development of PE). The fact that there is no evidence about IMA’s role in PE in the last 10 years highlights the need for more studies with greater sample sizes in order to conclude more reliable results. Furthermore, IMA may be elevated in many other conditions (e.g., exercise, congestive heart failure), thus performing multivariate analysis for possible confounders is crucial. Table 2 summarizes the available on the use of IMA in the diagnosis of PE.

## 5. Factor VIII

Factor VIII (FVIII) is a glycoprotein produced in liver sinusoidal cells and endothelial cells which is essential in the coagulation cascade [17]. Activated Factor VIII (FVIIIa) is derived by limited proteolysis, catalyzed by thrombin or activated factor X. FVIIIa increases the catalytic efficiency of activated factor IX in the activation of factor X. FVIII accelerates, through Xa, the conversion of prothrombin to thrombin, which converts fibrinogen to fibrin. Additionally, high FVIII levels may increase the thrombotic risk by decreasing responsiveness to activated protein C (APC) [18]. Consequently, FVIII plays an important role in the amplification of the clotting cascade at sites of vascular injury [19]. Studies indicate a direct relationship between high plasma levels of FVIII and arterial or venous thrombosis and PE [20,21,22,23,24,25,26,27,28,29,30].

Several studies reported that elevated plasma FVIII levels may represent a significant, independent risk factor for PE in a quantitative dependent pattern [20,21,23,24,26,27,28,29,30,31]. In 1995, Koster et al. reported the independent quantitative response association between FVIII levels and DVT (*p* < 0.001). In patients with FVIII levels above 1500 IU/L, there was a dose–response relation of FVIII levels with the risk of thrombosis (OR = 4.8) [20]. Other investigators also confirmed this independent and quantitative relationship. Rietveld et al. in 2019 [31], performed a large case–control study that assessed the levels of coagulation factors in relation to the risk for VTE. Their results showed that FVIII levels, as well as Von Willebrand Factor levels, had the strongest association of all coagulation factors with VTE, and this association was found to be independent of BMI, major illness, or CRP levels. The relative risks were similar for patients with provoked and unprovoked (idiopathic) VTE. The relative risks were found to be 15.0 (95% CI 8.6–26.1) for PE, 27.8 (95% CI 16.9–45.8) for DVT, and 43.2 (95% CI 16.6–122.5) for PE with DVT, suggesting that FVIII levels play different roles in the DVT and PE etiology [31]. Payne et al. also supported the independent relationship between FVIII levels and VTE [30]. In addition, it has been shown that Von Willebrand Factor levels correlate with FVIII levels, and this combination elevates, even more, the risk for VTE [24,30,31]. Interestingly, O’Donnell J et al. reported that elevated FVIII:C levels following VTE are persistent and independent of the acute phase reaction [23]. This result was further supported by Sane et al. who measured FVIII levels during PE diagnosis in 63 patients and after a 7-month follow-up period. The levels of FVIII were higher during PE diagnosis compared with the follow-up levels (167.2% vs. 155.1%) but the difference was not statistically significant (*p* = 0.07) [32]. Moreover, Oger et al. demonstrated a quantitative relationship between FVIII levels and VTE, not only in young adults but elderly patients (>70 years old) as well [26].

Regarding PE, Erkekol et al. supported the existence of a quantitative correlation between factor VIII levels and thrombosis, since high plasma levels of FVIII (>168 U/dL) were found in 53.3% (OR 11.04; 95% CI 3.65–33.35) of isolated PE patients and 55.0% (OR 11.81; CI 3.49–39.92) of patients with a combined form of PE and DVT compared with 9.4% in control patients. The risk was not affected after adjustment for other possible risk factors [27]. Heerink et al. found that the levels of FVIII were higher in patients with PE when compared with healthy controls, but the population of PE patients in this study was small (*n* = 11) [33]. In contrast to the previous results pointing to the specificity of FVIII levels, Kamphuisen et al. found high FVIII levels (≥150 IU/dL) at the acute phase in both PE patients and subjects with various etiologic substrates (pneumonia, heart failure, or malignancy) [25].

While several studies have investigated the possible use of FVIII levels as a biomarker for the diagnosis of PE, their importance remains unclear. The aforementioned observations need further confirmation by studies focusing on the potential influence of comorbidities (such as cancer, heart disease, or lung disease) and the acute phase reactions at the FVIII plasma level.

## 6. MicroRNAs

MicroRNAs (miRNAs) are non-coding RNAs with a length of approximately 22 nucleotides that are involved in important cellular pathways such as development, proliferation, and apoptosis [34]. They are present in various body fluids, being remarkably stable due to carrier-protein binding [35] and consequently, in recent years, they have been studied as non-invasive biomarkers for various disorders such as cancer, cardiovascular, and cerebrovascular diseases [36,37]. Various miRNAs have been reported to regulate several hemostatic factors (fibrinogen, factor XI, etc.), modulate platelet activation and aggregation, and have been found to be dysregulated in venous thrombosis [38,39].

In 2011, Xiao et al. were the first group that investigated the possibility of identifying some miRNAs as potential biomarkers for the diagnosis of acute PE. Specifically, they reported that miRNA-134 was significantly elevated in patients with acute PE when compared with healthy individuals, reporting a sensitivity of 68.8% and specificity of 68.2% [40]. In 2016, Deng et al. [41] published a systematic review and meta-analysis based on three studies [40,42,43], concluding that, although more research is needed to validate their role as diagnostic biomarkers for acute PE, miRNAs seem to represent reliable novel biomarker candidates (pooled sensitivity: 83%, pooled specificity: 85%). Since then, more original studies have been published [44,45,46], reporting that various miRNAs are upregulated during acute PE episodes and can be an additional tool available to the physician. An interesting study reported that miRNA-1233 and miRNA-134 can be potentially used to identify patients with acute exacerbation of chronic obstructive pulmonary disease complicated by acute PE [46].

It is important to note that some groups have investigated the possibility of combining various miRNAs with each other and with other established biomarkers such as D-dimers to increase their diagnostic efficacy [45]. Combining miRNA-27a/b with D-dimers significantly increases the capacity for diagnosing acute PE [45]. In more detail, combining miRNA-27a or miRNA-27b with D-dimers resulted in a significant increase in the area under the receiver operating characteristic (ROC) curve, reaching 0.909 and 0.867, respectively.

Collectively, these findings support the hypothesis that miRNAs may serve as a novel biomarker for the diagnosis of acute PE; however, further research in this field has to be performed, as studies up to this date have limited statistical power and reproducibility. Table 3 summarizes the available data on the use of miRNAs in the diagnosis of PE.

## 7. Microparticles

Microparticles (MPs) are small vesicles of <1 micron in size, derived from various cell types, including platelets, monocytes, endothelial, and cancer cells [5,47]. MPs are formed from membrane vesicles released from the cell surface by the proteolytic cleavage of the cytoskeleton. MPs provide procoagulant molecules, especially anionic phospholipids (particularly phosphatidylserine) and tissue factor (TF) protein, for the assembly of components of the coagulation cascade [47]. As a result, many studies aimed to investigate the role of MPs in inflammation, cancer, and other diseases [48]. Due to their involvement in the formation of thrombi, MPs have been proposed as potential biomarkers for the diagnosis of VTE and acute PE [48,49].

In a study by Rezania S et al. plasma MP levels were evaluated in PE patients and healthy volunteers using standard fluorescent polystyrene beads. A relative abundance of plasma MPs was noted in the PE patients [50]. Furthermore, there was a correlation between the PE and Platelet-Derived MPs (PDMPs) plasma levels; the PE patients exhibited higher levels of PDMPs as compared to their healthy counterparts [51]. The association between acute PE and procoagulant MPs levels was also highlighted by Bal et al. [52]. Specifically, circulating MPs and platelet MPs were significantly elevated in PE patients compared to healthy controls, but this correlation was not statistically significant when PE patients were compared with controls with cardiovascular risk factors such as hypertension and diabetes [52].

The correlation between MP levels and cardiovascular diseases, such as hypertension and coronary artery disease, has been previously reported [52,53]. These results highlight the importance of adjusting the measurement of MPs in patients with cardiac comorbidities when PE is suspected. 

In conclusion, these studies provide evidence in favor of the clinical use of MPs for diagnosis, but potentially after adjustment for other comorbidities (cancer, cardiovascular diseases) which may also increase the levels of MPs in patients’ serum.

## 8. Other Biomarkers and PE

There are several more novel biomarkers that have been proposed as potential biomarkers for the diagnosis of PE. Limited data on those biomarkers are available; thus, their use in clinical practice is still considered questionable.

Endothelial cell-specific molecule 1, or endocan, a soluble dermatan sulfate proteoglycan [54], has been associated with PE, predicting the severity of pulmonary artery occlusion [55,56]. Güzel et al. measured serum endocan levels in 46 PE patients and reported a significant difference in the serum endocan levels between PE patients and healthy controls 321.93 vs. 192.77 mg/L (*p* < 0.03) [55]. On the contrary, Mosevoll et al. dispute the diagnostic value of endocan in PE, showcasing that no difference in endocan levels between patients with PE and healthy controls was noted in their study [57]. Thus, the use of endocan as a PE biomarker has to be examined further before definite conclusions can be drawn.

The soluble urokinase-type plasminogen activator receptor (suPAR) is the soluble form of the urokinase-type plasminogen activator receptor, which is a glycosyl-phosphatidylinositol (GPI)-linked membrane protein [58] and an integral part of the fibrinolytic system. SuPAR has also been related to VTE. The incidence of VTE in 5203 subjects was higher in patients with high suPAR levels independently of several potential confounding factors (age, sex, BMI, smoking, systolic blood pressure, cholesterol, HDL, leukocyte count, diabetes, history of atrial fibrillation, history of cardiovascular disease) during a 15.7-year follow-up [58]. However, suPARs’ role in PE is still unclear and needs further examination before definite conclusions can be drawn.

C-reactive protein (CRP) is a biomarker of systemic inflammation which has also been evaluated as a diagnostic marker in PE. CRP levels had a sensitivity of 95.7% [95% confidence interval (CI): 90–100] and an NPV of 98.4% (96–100). CRP < 5 mg/L with a clinical probability score indicating ‘PE unlikely’, had a sensitivity of 96.7% (90–100), specificity of 43.0% (37–49), and NPV 99.1% (97–100) [59]. Moreover, lower CRP levels have been shown to relate to unprovoked (idiopathic) PE [60], and changes in serum high-sensitivity CRP (Hs-CRP) levels could help in the severity categorization of PE (i.e., massive or minor PE) patients, as well as outcome prediction [61]. Thus, it has been proposed that CRP levels could be used to safely exclude PE, either alone or combined with clinical probability assessment [59,61].

A contemporary working hypothesis suggests that proteomic analysis could be used to identify potential biomarkers for PE. According to Granholm F et al. [62], the use of proteomic analysis showed that Complement component 9, Complement factor H, and Leucine-rich α-2-glycoprotein were increased in patients with PE, whereas Carboxylic ester hydrolase, Antithrombin-III, Procollagen C-endopeptidase enhancer, Serpin peptidase inhibitor, clade A, member 4, Afamin, and, *N*-acetylmuramoyl-l-alanine amidase, among others, were decreased. These findings consider proteins of general inflammation, atherosclerosis, and hemostasis as possible biomarkers in the diagnosis of PE while further research is needed to confirm it [62]. In the same context, data acquisition mass spectrometry and antibody microarray studies revealed that serum amyloid A1, calprotectin, and tenascin-C have promising value in PE diagnosis with acceptable sensitivity and specificity [63]. Few data support the use of haptoglobin (a hemoglobin-binding protein that serves as an acute-phase protein) as a biomarker of PE since its levels are increased in PE patients; the cut-off of 256.74 mg/L had a sensitivity of 62% and specificity of 83% for PE diagnosis [64].

## 9. Cost-Effectiveness Perspectives

Another significant parameter regarding the biomarkers in PE diagnosis is the evaluation of cost-effectiveness in health care systems worldwide. In fact, the use of an age-adjusted D-dimer cutoff of <500 ng/mL up to 50 years, then <age × 10 ng/mL, increases the cost savings by more than USD 80 million per year for the United States health care system [65]. Moreover, the D-dimer test and lower-limb compression ultrasonography are not only cost-effective in the diagnosis of PE but also easily available, thus allowing centers devoid of CTPAs to screen patients with suspected PE and avoid costly referrals [66]. On the other hand, novel biomarkers are usually expensive, and in an increasingly cost-conscious health care environment, regulatory approval will not be a guarantee of clinical adoption. Researchers must prove that a specific biomarker can change clinical practice and reduce costs by eliminating time-consuming, expensive, and sometimes ineffective diagnostic tests.

Table 4 summarizes the available data on the use of novel biomarkers in the diagnosis of PE, according to our literature search.

## 10. Conclusions

There is evidence supporting the use of biomarkers to enhance the diagnosis and predict the recurrence risk in patients with PE. There are several molecules related to both thrombotic and inflammatory PE-associated processes under investigation. So far, only D-dimer levels have been widely employed for the diagnosis of PE in daily clinical practice and have been implemented in international guidelines for PE diagnosis. This review was conducted in order to shed more light on non-D-dimer biomarkers, such as fibrinogen, IMA, factor VIII, microRNAs, and microparticles, which are present in the current bibliography, even though there are not yet randomized control trials to confirm and advance the existing evidence. Major caveats that should be addressed before the incorporation of biomarkers in clinical decision algorithms include the estimation of optimal cut-off values, the need for adjustment for cofounders (i.e., renal function), and the absence of studies that perform external validation. Further studies addressing the role of the aforementioned candidate biomarkers in PE should be designed to elucidate their importance in the diagnostic algorithm of the disease.

## Figures and Tables

**Table 1:** Summary of the available studies on the use of fibrinogen in the diagnosis of PE [4,6,8,10].

Study/YOP	Number of Participants	Results
Kucher et al., 2003 [6]	191	A D/F ratio of >103 is highly specific for the presence of acute PE It doubles the diagnostic rate compared with D-dimer testing alone
Kara et al., 2014 [4]	200	D-dimer cutoff of 0.5 mg/mL vs. D/F ratio cutoff of 1.0: D/F ratio may have a better specificity than D-dimer level in PE diagnosis
Hajsadeghi et al., 2012 [10]	81	Significantly higher D/F ratio (0.913 ± 0.716 vs. 483 ± 0.440 × 10^−3^, *p* = 0.003) in PE patients than in non-PE patients. A D/F ratio of 0.417 × 10^−3^ (AUC = 0.710, *p* = 0.004) had 70.3% sensitivity and 61.6% specificity.
Calvo-Romero et al., 2004 [8]	40	Fibrinogen levels similar in patients with a negative vs. positive D-dimer test Fibrinogen levels not statistically different in patients with DVT and PE vs. patients with isolated DVT (trend observed, *p* = 0.1).

**Table 2:** Summary of the available studies on the use of IMA in the diagnosis of PE [12,16].

Study/YOP	Number of Participants	Results
Turedi et al., 2006 [16]	60	Mean IMA levels in PE patients: 0.724 ± 0.122 ABSU Mean IMA levels in controls: 0.360 ± 0.090 ABSU Statistically significant difference (*p* < 0.0005)
Turedi et al., 2008 [12]	189	Cut-off point of 0.25 ABSU: Sensitivity = 93% Specificity = 75% PPV = 79.4% NPV = 78.6% For PE diagnosis

**Table 3:** Summary of the available studies on the use of miRNAs in the diagnosis of PE [40,42,43,44,45,46].

Study/YOP	No Subjects/ Sample from	Cut-Off Value	Molecule	Results
Xiao J et al., 2011 [40]	54/plasma	0.003	miRNA-134	miRNA-134 levels were higher in patients with acute PE compared with healthy individuals, reporting a sensitivity of 68.8% and specificity of 68.2% Additionally, used miRNA-134 to differentiate between acute PE patients and non-PE patients that reported dyspnea, chest pain, or cough
Zhou X et al., 2016 [42]	74/plasma	1.66	miRNA-28-3p	MiRNA-28-3p was significantly elevated in the plasma of PE patients.
Kessler et al., 2016 [43]	42/plasma	0.53 0.63 0.51	miRNA-1233, miRNA-27a, miRNA-134	miRNA-1233, miRNA-27a, and miRNA-134 were significantly higher in the serum of acute PE patients in comparison to healthy controls The 1233-miRNA differentiated the acute PE patients and the NSTEMI, DVT, and chronic pulmonary hypertension patients
Liu T et al., 2018 [44]	110/plasma	NA	miRNA-221	The plasma levels of miRNA-221 were significantly increased in patients with acute PE when compared with healthy individuals. The levels in patients with acute PE were positively correlated with levels of BNP, troponin I, and D-dimer.
Wang Q et al., 2018 [45]	148/plasma	0.115, 0.059	miRNA-27a miRNA-27b	The plasma levels of miRNA-27a and miRNA-27b were significantly higher in APE patients (*p* < 0.001) compared with normal controls. Combining miRNA-27a/b with D-dimers significantly increased the capacity for diagnosing acute PE
Peng L et al., 2020 [46]	52/plasma	NA	miRNA-1233, miRNA-134	miRNA-1233 and miRNA-134 have high clinical value in the early diagnosis of patients of acute exacerbation of chronic obstructive pulmonary disease combined with PE These miRNAs could be used as potential biomarkers for clinical identification of acute exacerbation of chronic obstructive pulmonary disease with or without PE complications.

**Table 4:** Summary of the use of novel biomarkers in the diagnosis of PE.

Biomarker	Comments
Fibrinogen	Fibrinogen as a biomarker itself seemed to be unreliable for PE diagnosis. D-Dimer/Fibrinogen ratio had promising results, especially for critically ill patients, such as ICU patients. Further larger randomized clinical trials have to confirm these findings.
Ischemia modified albumin	IMA levels in combination with clinical probability scores (Geneva and Wells score) seem to play a role in the diagnosis of PE. Τhere is a lack of evidence in the last 10 years and a small number of available studies.
Factor VIII	The correlation between FVIII and PE has been proven by a few studies over the last three decades. Evidence in favor of specificity of FVIII in PE diagnosis. Data supports that FVIII is independent of patients’ comorbidities. Other studies report that FVIII is high in various other disease processes. Despite the promising evidence in favor of the FVIII in PE diagnosis, its specificity and accuracy are still questioned.
MicroRNAs	Several miRNAs (miRNA-1233 and miRNA-134) are upregulated during acute PE episodes, making them potentially useful in PE diagnosis. MiRNA-27a/b in combination with D-dimers significantly increases the capacity for diagnosing acute PE.
Microparticles	MPs, especially platelet-derived, were found to be elevated in PE patients. However, MPs also increase in other cardiovascular diseases. This points out the need for adjusting the measurement of MPs in patients with cardiac comorbidities

## Data Availability

Not applicable.
